# Adaptive Threonine Increase in Transmembrane Regions of Mitochondrial Proteins in Higher Primates

**DOI:** 10.1371/journal.pone.0003343

**Published:** 2008-10-06

**Authors:** Yasuhiro Kitazoe, Hirohisa Kishino, Masami Hasegawa, Noriaki Nakajima, Jeffrey L. Thorne, Masashi Tanaka

**Affiliations:** 1 Center of Medical Information Science, Kochi Medical School, Nankoku, Kochi, Japan; 2 Graduate School of Agricultural and Life Sciences, University of Tokyo, Yayoi, Bunkyo, Tokyo, Japan; 3 School of Life Sciences, Fudan University, Shanghai, China; 4 Bioinformatics Research Center, North Carolina State University, Raleigh, North Carolina, United States of America; 5 Department of Genomics for Longevity and Health, Tokyo Metropolitan Institute of Gerontology, Tokyo, Japan; Max Planck Institute for Evolutionary Anthropology, Germany

## Abstract

**Background:**

The mitochondrial (mt) gene tree of placental mammals reveals a very strong acceleration of the amino acid (AA) replacement rate and a change in AA compositional bias in the lineage leading to the higher primates (simians), in contrast to the nuclear gene tree. Whether this acceleration and compositional bias were caused by adaptive evolution at the AA level or directional mutation pressure at the DNA level has been vigorously debated.

**Methodology/Principal Findings:**

Our phylogenetic analysis indicates that the rate acceleration in the simian lineage is accompanied by a marked increase in threonine (Thr) residues in the transmembrane helix regions of mt DNA-encoded proteins. This Thr increase involved the replacement of hydrophobic AAs in the membrane interior. Even after accounting for lack of independence due to phylogeny, a regression analysis reveals a statistical significant positive correlation between Thr composition and longevity in primates.

**Conclusion/Significance:**

Because crucial roles of Thr and Ser in membrane proteins have been proposed to be the formation of hydrogen bonds enhancing helix-helix interactions, the Thr increase detected in the higher primates might be adaptive by serving to reinforce stability of mt proteins in the inner membrane. The correlation between Thr composition in the membrane interior and the longevity of animals is striking, especially because some mt functions are thought to be involved in aging.

## Introduction

**Figure 1:**
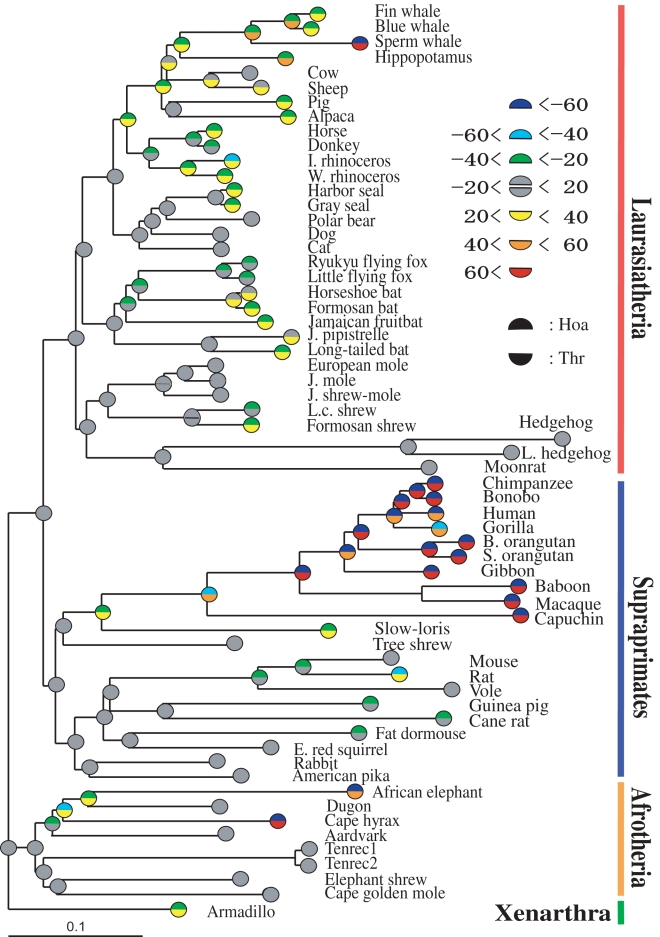
Hoa→Thr flows on a placental mammal tree. The tree was reconstructed with a multidimensional vector space (MVS) method from 62 complete mt AA sequences [2]. The upper and lower half circles on the ancestral nodes and terminals represent the numbers of Hoa and Thr, respectively, relative to those of the root sequence. The decrease of Hoa and the increase of Thr were expressed by gradational changes from dark colors toward light ones of four grades (see the insertion). We defined the root sequence as the most recent common ancestor of Afrotheria and Xenarthra, and estimated the node sequences by applying the maximum parsimony algorithm to three groups of Hoa, Thr and Rma (the remaining AAs except for Hoa and Thr) (Materials and Methods).

Mitochondria supply most cellular energy and influence cell growth, human disease, and probably aging [1]. A better understanding of mt protein functions may be possible by detecting adaptive evolution that occurred in specific lineages of the mt gene tree of the placental mammals. Interestingly, the mt gene tree displays elevated rates of AA replacement in lineages leading to the simians, rodents, and hedgehogs (Figure 1) [2]. Although the nuclear gene tree also shows rate accelerations in the lineages of rodents and hedgehogs, no significant acceleration is observed in the primate lineage [3], [4] whereas the mt branch lengths of the simians (relative to the total branch lengths) are twice as long as those on the nuclear gene tree. This feature of the mt gene tree suggests that the simian lineage experienced a particularly unusual event in molecular evolution. One hypothesis is that an episode of adaptive evolution occurred in the simian lineage [5]–[9]. Another hypothesis is that the acceleration and compositional bias were caused by a higher rate of directional mutation [10], [11]. The adaptive hypothesis is supported by a highly significant acceleration of nonsynonymous changes, in contrast to a rather homogeneous rate of synonymous changes. The mutation bias explanation is supported by the existence of directional nucleotide mutation pressure at the fourfold-degenerate third nucleotide sites (FD3rd) in codons [12]–[14], and also by the duration of the single-stranded state of the 12 mt protein-coding genes (except for NADH dehydrogenase subunit 6) during replication [12].

Here, we find that the rate acceleration in the simian lineage is accompanied by a marked increase in Thr residues within the transmembrane region of mt proteins. This Thr increase may be adaptive because it is correlated with the protein structure. An essential role of Thr residues is to stabilize mt proteins by forming helix-helix interactions [15]–[19]. We also find an intriguing evolutionary correlation between the increase in maximum life span and the increase in Thr composition of membrane interiors.

## Results

### Decrease in hydrophobic AAs and increase in Thr residues on the placental mammal tree

We first report that the 12 mt protein-coding genes underwent, at an early stage of simian evolution, a net flow of AA replacements from hydrophobic AAs (Hoa) to Thr. We refer to this compositional change as the Hoa→Thr flow. For this flow analysis, we classify eight amino acid types (Ala, Cys, Ile, Leu, Met, Phe, Pro and Val) as hydrophobic (Table S1). The Hoa→Thr flow violates the “detailed balance” condition that would have the numbers of AA replacements from Hoa to Thr be about equal to that from Thr to Hoa. Figure 1 plots the numbers of Hoa and Thr residues (relative to those of the root sequence) in each inferred node sequence on a placental mammal tree [2]. The Hoa→Thr flow appeared specifically in the long branches leading to the simians, as well as in those leading to the sperm whale and elephant. However, the flow did not occur in the long branches of the mt or nuclear gene trees leading to the rodents or hedgehogs.

### Hoa→Thr flow in the higher primate lineage

**Figure 2:**
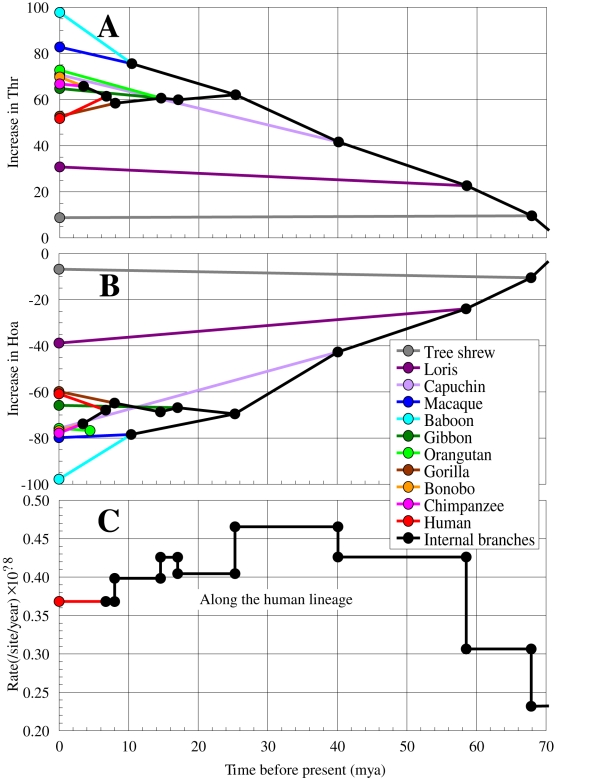
Hoa→Thr flow along the primate lineage. Figures A, B and C represent, respectively, the increased number in Thr, the decreased number in Hoa (relative to the Thr/Hoa numbers of the root sequence) and the evolutionary rate, as functions of the divergence times of primates. The evolutionary rate was traced along the human lineage. The values of the times and rates were estimated using a harmonic mean method which is robust against strong evolutionary rate changes [20]. Here, we used the root and node AA sequences which were obtained in Figure 1.

By chronologically tracing the greatest flow along the simian lineage, we detect a strong correlation between the decrease in Hoa and the increase in Thr (Figure 2**A** and **B**). The tree shrew lineage, with a very short branch (Figure 1), had a minimal flow, and the loris lineage, with a moderately long branch, had a small flow. We also observe that the Hoa→Thr flow is associated with an accelerated AA evolutionary rate (Figure 2**C**) in the early stages of primate evolution previously reported [20]. This observation seems to be consistent with the higher rates of nonsynonymous relative to synonymous substitutions that occurred in complexes IV (cytochrome c oxidase subunits) [5]–[9]. By applying the flow analysis to each protein, we found that the Hoa→Thr flow was generally large in complexes I (NADH dehydrogenase subunits 1, 2, 4 & 5; ND1, ND2, ND4 & ND5) and III (cytochrome b; Cytb), and rather small in complexes IV (cytochrome c oxidase subunits) and V (ATPase subunits). We note that ND2 displayed a Hoa→Ser flow in addition to the Hoa→Thr flow.

### Hydrophobicity dependence of Hoa→Thr flow

**Figure 3:**
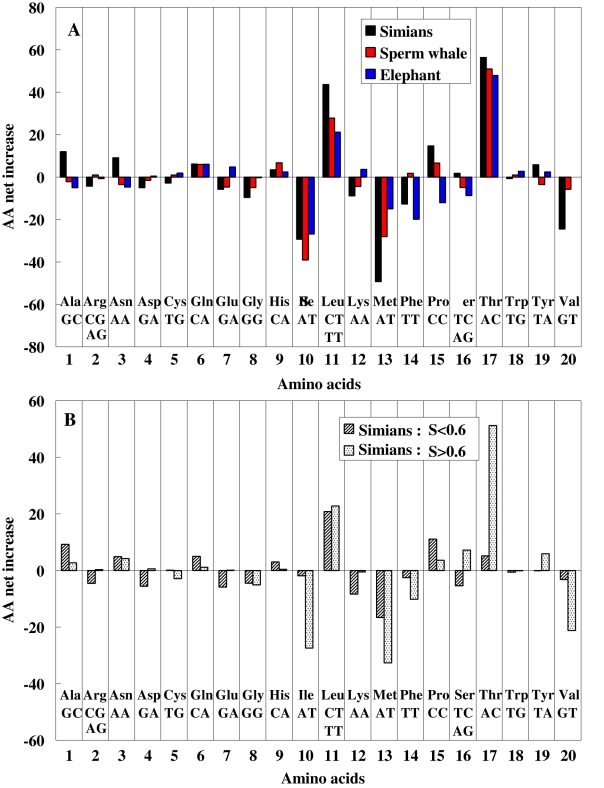
AA transition net flows in the three lineages of simians, sperm whale, and elephant, and hydrophobicity dependence. Figure A represents the net flow of each AA in the three lineages of the primate, the sperm whale and the elephant. The net flow of an AA is the site number of this AA which increased or decreased in terminal sequences, relative to a root sequence. The roots of the primates, the sperm whale and the elephant were defined as the most recent common ancestors of the tree shrew and primates, the sperm whale and hippopotamus, and the elephant and golden mole, respectively (Figure 1). The bottom lines denote the 20 AAs and the nucleotides at the first and second codon sites. Figure B represents the hydrophobicity dependence of the AA net flows in the two regions *S*<0.6 and *S*>0.6 of mt proteins, for the simian lineage (Methods and Materials) and shows that the Hoa →Thr flow appears in only the AA sites with *S*>0.6 while the increases of the Leu residues exists independent of the *S* values because of the directional mutation pressure causing the T→C flow. *S* expresses the local hydrophobic environment of AA sequences and is given by a moving average of local hydrophobic scores around each AA site (Materials and Methods).

**Figure 4:**
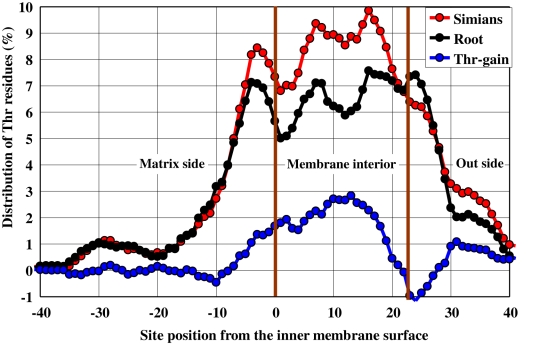
Thr distribution measured from the membrane surface on the matrix side. Figure estimates the spatial distribution of Thr residues in the 12 mt proteins. The *x*-values stand for the AA site numbers measured from the inner membrane surface on the matrix side. The inner and outer surface positions (vertical lines) of the helices in each protein AA sequence were inferred from a secondary structure analysis of the mt proteins [21]–[23]. The black and red circles show the Thr compositional distribution (%) in the root sequence (the most recent common ancestor of the tree shrew and primates) of the primates and that (the averaged value) in the simian terminal sequences, respectively. The blue circles represent the increased fraction (gain) of Thr residues in the simians.

As described in Materials and Methods, we analyzed in more detail the Hoa→Thr flow in the three lineages of simians and also sperm whale and elephant, by locally estimating the node sequences of the three lineages at the DNA level. The analysis displayed both substantial increases in Thr/Leu residues and reductions in Ile/Met residues (Figure 3**A**). The increase in hydrophobic Leu residues accompanied a compensatory decline in other hydrophobic residues. Consequently, only the Hoa→Thr flow appeared. We next performed a protein structure analysis [21]–[23], by introducing a moving average, *S*, of local hydrophobic scores around each AA site (Materials and Methods), and expressed the local hydrophobic environment of the root sequences using an AA scale model (Table S1 gives the hydrophobic score of each AA with an orthodox model [24]). We find that the Hoa→Thr flow occurred especially in the hydrophobic region (*S*>0.6) of mt proteins (the frequency distribution of *S*-values gives the maximum at *S* = 0.6, whereas the remaining 11 AAs (Rma) except for Hoa and Thr had no flow (Figure 3**B** and Table 1**A**). This hydrophobicity dependence of the Hoa→Thr flow was also observed in the sperm whale and elephant lineages (Table 1**B** and **1C**). To investigate this dependence in detail, we estimated the spatial distribution of the Thr residues around the membrane interior using a transmembrane analysis to predict the helical regions of the AA sequences [22], [23]. Here, the distribution was estimated in the simian and root sequences. It was measured from the AA coordinates of the membrane surface on the matrix side by splitting an AA sequence into fragments, each of which was embedded only once in the membrane interior. Figure 4 demonstrates that the Thr increase in the lineage leading to the simians took place preferentially in the membrane-spanning (helix) regions. Because the maximum parsimony reconstruction of the root sequence may be biased toward the consensus state [25] and this could produce an artifact that incorrectly suggests an AA composition flow, we conducted an additional analysis with the simian lineage. We defined the root of the simians as the most recent common ancestor of the tree shrew and the primates. Then, the root sequence estimated could be approximately substituted by the real sequence of the tree shrew, because the branch length between the root and the tree shrew is very short, as seen in Figure 1. This substitution was useful to confirm that the estimated root sequence is not biased, and provided a similar result of the Hoa→Thr flow to that given by the estimated root sequence (Tables 1**A** versus 1**D**). Table S2 lists the AA positions of mt proteins at which the Hoa sites of the primate root sequence in the hydrophobic region (*S*>0.6) changed to Thr in chimpanzee or human.

**Table 1:** Hoa→Thr flow in the three lineages of simians, sperm whale, and elephant, and hydrophobicity dependence.

	S<0.6		S>0.6		Flow
	In	Out	In	Out	
**A. Simians**					
Hoa	83	75	**33**	**102**	**−61**
Thr	70	67	**76**	**24**	**55**
Rma	68	79	39	22	6
**B. Sperm whale**					
Hoa	35	44	**19**	**49**	**−39**
Thr	40	25	**45**	**16**	**44**
Rma	26	32	13	12	−5
**C. Elephant**					
Hoa	75	66	**33**	**93**	**−51**
Thr	59	60	**75**	**23**	**51**
Rma	69	76	33	26	0
**D. Simians (tree shrew)**					
Hoa	95	104	**40**	**98**	**−66**
Thr	105	90	**79**	**33**	**61**
Rma	93	100	38	26	5

Table gives the incoming (In) and outgoing (Out) AA numbers for the three AA groups of Hoa, Thr, and Rma (the remaining AAs except for Hoa and Thr), between the root and terminal sequences. For example, in the case **A**, the In and Out numbers denote the average site numbers of Thr residues which appeared and disappeared in the simian terminal sequences, respectively. These numbers were estimated in the two AA site domains, *S*<0.6 and *S*>0.6, of the local hydrophobic environment values (Methods and Materials). The flow denotes the differences of the AA numbers between In and Out, which become quite large in the high *S*-value domain of the AA sites. **A**, **B** and **C** represent the three cases of simians, sperm whale, and elephant, respectively. **D** gives the case of the real sequence of the tree shrew with a short branch from the root, to confirm that the estimation of the root sequence is not biased. The roots of the simians, the sperm whale and the elephant were defined as the most recent common ancestors of the tree shrew and primates, the sperm whale and hippopotamus, and the elephant and golden mole, respectively (Figure 1).

### Discrimination between adaptive evolution and mutation bias at the DNA level

By estimating the node sequences at the nucleotide level, we calculated the numbers of nonsynonymous changes at the first and second codon sites, and also the number of synonymous changes at the FD3rd codon sites, between the root sequence and the terminal sequences. A predominant T→C flow (T, thymine; C, cytosine) at the second codon site was inferred in the three lineages of simians, sperm whale, and elephant (Tables 2**A**–2**C**). Furthermore, this flow occurred preferentially in the highly hydrophobic regions, and produced the main part (the Ile/Met→Thr flow) of the Hoa→Thr flow. We also note that the T→C flow direction was reversed at the FD3rd codon sites of the sperm whale and elephant lineages, with no hydrophobicity dependence. Conversely, the predominant A→C flow (A, adenine) was inferred at the FD3rd codon sites, without hydrophobicity dependence in the simian and sperm whale lineages. The A→C flow also occurred at the first codon sites and yielded intraflows (the Ile/Met→Leu) among the Hoa, but the increase in Leu residues showed no hydrophobicity dependence, in contrast to the increase in Thr residues (Figure 3**B**). Consequently, we conclude that the T→C flow was induced by AA adaptive evolution, whereas the A→C flow was caused by nucleotide mutation bias. This conclusion was supported by using again the tree shrew instead of the primate root (Tables 2**A** versus 2**D**), and by estimating the significance of differences between two nucleotide flows in the hydrophobic regions of *S*<0.6 and *S*>0.6. The T→C flow for adaptive evolution in the simian lineage showed a significant hydrophobicity dependence with p = 1.123×10^−5^.

**Table 2:** Nucleotide substitution flows in the three lineages of simians, sperm whale, and elephant.

	1st	2nd	FD3rd
**A. Simians**			
T→C	17 (10,7)	**67 (6,61)**	19 (10,9)
T→A	6 (5,1)	6 (−2,8)	−51 (−28,−23)
T→G	−1 (2,−3)	0 (0,0)	2 (2,0)
A→C	**48 (25,23)**	3 (4,−1)	**196 (99,97)**
G→C	4 (1,3)	8 (3,5)	7 (4,3)
G→A	28 (10,18)	4 (1,3)	−41 (−20,−21)
**B. Sperm whale**			
T→C	0 (−1,1)	**37 (12,25)**	−35 (−20,−15)
T→A	−3 (2,−5)	0 (−4,4)	−56 (−25,−31)
T→G	−2 (−3,1)	0 (1,−1)	5 (3,2)
A→C	**27 (14,13)**	3 (4,−1)	**103 (38,65)**
G→C	11 (6,5)	5 (1,4)	0 (−1,1)
G→A	9 (5,4)	2 (1,1)	−50 (−25,−25)
**C. Elephant**			
T→C	18 (13,5)	**29 (−7,36)**	−19 (−7,−12)
T→A	−1 (−3,2)	7 (4,3)	−37 (−13,−24)
T→G	−6 (−2,−4)	4 (1,3)	2 (2,0)
A→C	10 (1,9)	−6 (−4,−2)	21 (21,0)
G→C	−8 (−5,−3)	−3 (−1,−2)	−8 (−6,−2)
G→A	−5 (−16,11)	−2 (−2,0)	−31 (−16,−15)
**D. Simians (tree shrew)**			
T→C	20 (12,8)	**49 (6,63)**	33 (12,21)
T→A	18 (10,8)	−1 (−7,7)	2 (−7,9)
T→G	−8 (−4,−4)	0 (0,0)	−4 (−4,0)
A→C	**48 (21,27)**	−3 (0,−3)	**134 (72,62)**
G→C	14 (7,7)	13 (4,9)	23 (12,11)
G→A	**71 (31,40)**	12 (7,5)	1 (3,−2)

Table gives the nucleotide substitution flows of nonsynonymous changes at the first and second codon sites, and those of synonymous changes at the FD3rd sites. The quantity X→Y denotes the net flow from X to Y. The values *u* and *v* in parentheses (*u*, *v*) denote the fractions in the two score regions *S*<0.6 and *S*>0.6, respectively. **A**, **B**, and **C** represent the three cases of simians, sperm whale, and elephant, respectively. **D** indicates the case of the real sequence of the tree shrew with a short branch instead of the root sequence of the primates, to confirm that the estimation of the root sequence is not very biased.

### Relationship between Thr increase and longevity in primates

**Figure 5:**
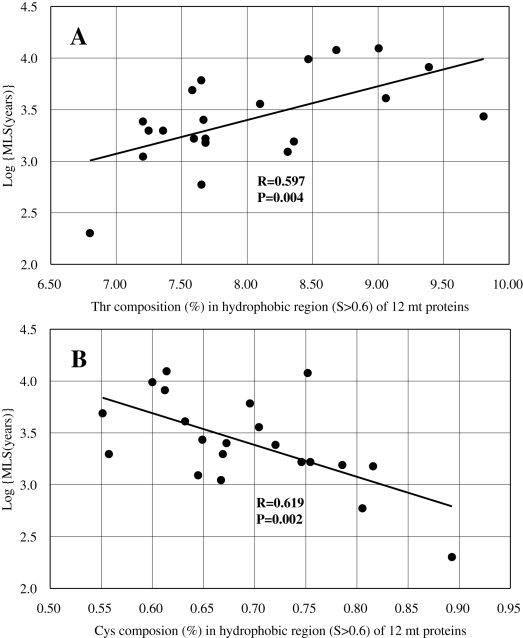
Correlation between the Thr/Cys compositions and the MLS in primates. The Thr-Log(MLS) and Cys-Log(MLS) correlations were estimated in the hydrophobic region (S>0.6) of 12 mt proteins (Table 3 includes the results of the phylogenetic generalized least-squares for the regression analysis). The insertion gives the correlation coefficients (R) and the P-values for significance. The magnitude (R = 0.597, P = 0.004) of the Thr-Log(MLS) correlation (Figure A) was compatible with that (R = 0.619, P = 0.002) of the Cys-Log(MLS) (Figure B). Human was excluded in the analysis, since it became an outlier with a larger deviation than 3σ from the regression line (σ; the standard deviation) and indeed the MLS value (122.5 years) of human seems to be too large.

We showed that the frequency of Thr residues greatly increased in the highly hydrophobic region (S>0.6) of mt proteins in the simians (Figure 3**B** and Table 1**A**). We speculate that this threonine increase may be related to life span via an increased stability of these proteins. Recently, Moosmann and Behl noted that Cys composition is negatively correlated with life span in a wide range of animals [26]. They argue that Cys depletion may render mt proteins more resistant to oxidative attack. By adding several taxa recently stored in the NCBI database [http://www.ncbi.nlm.nih.gov/Genbank/] and then calculating the Cys and Thr compositions (%) of primate AA sequences, we characterized the correlations between these AA compositions and the logarithm of maximum life spans (MLS). This was done by using phylogenetic generalized least-squares [27] because it takes into account the effect of tree structure within a multivariate regression framework (Materials and Methods; Table 3). A significant relationship between Thr composition and the logarithm of MLS appeared in primates where a predominant Hoa→Thr flow is observed (Figure 5**A**). Interestingly, the magnitude of this correlation was compatible with that of the Cys-Log (MLS) negative correlation (Figure 5**B**). The correlation was stronger in the hydrophobic region (*S*>0.6) of 4 proteins (ND1, ND2, ND4 and Cytb). Table S3 gives the values of Thr/Cys compositions and Log (MLS) in primates and the accession number of mt DNA sequences used in this analysis.

**Table 3:** Thr & Cys-MLS correlations in 20 primates and tree shrew.

	12 proteins				4 proteins	
	AA sites (all)		AA sites (S>0.6)		AA sites (S>0.6)	
	LS	PGLS	LS	PGLS	LS	PGLS
Thr	0.431	0.492	0.597	0.439	0.568	0.533
	(0.051)	(0.025)	(0.004)	(0.047)	(0.007)	(0.013)
Cys	0.575	0.458	0.619	0.467	0.523	0.53
	(0.006)	(0.038)	(0.002)	(0.034)	(0.016)	(0.013)
Thr+Cys	0.607	0.552	0.740	0.535	0.650	0.656
	(0.015)	(0.030)	(0.001)	(0.035)	(0.007)	(0.007)

We estimated the Thr-Log(MLS), Cys-Log(MLS) and Thr/Cys-Log(MLS) correlation coefficients (R) by using the multivariate regression analysis. The result of least square (LS) which does not take account of the tree structure is compared with that of the phylogenetic generalized least-squares (PGLS) which takes account of it (Materials and Methods). The parentheses give the p-values for significance. The correlations were more explicit in the hydrophobic region (S>0.6) of the AA sites in the 4 proteins (ND1, ND2, ND4 and Cytb).

## Discussion

The compositional changes that we have detected in the transmembrane regions may be of importance to those who are using mitochondrial DNA sequences for phylogenetic studies, because standard models of sequence analysis do not permit or predict these changes. The correlation at the AA level between the Thr composition in the hydrophobic region and the MLS in primates is intriguing, since many other factors to regulate longevity may be considered at a macroscopic level [32] and a recent study reports that the base composition of mtDNA, especially the G or GC content could undergo co-evolution with the mammalian longevity [33].

Schmitz et al. pointed out a compositional nucleotide shift from T→C and A→C in the higher primates, and demonstrated the impact of these changes on the AA composition [13]. Furthermore, Gibson et al. suggested that the increase in Thr residues in 12 mt proteins correlated with the increase in the C content [14]. These authors argued that the variations in the AA compositions among species were caused by nucleotide mutation bias. However, those studies were mainly based on inclusive compositional regression analysis of the terminal sequences. We investigated the nucleotide changes along the evolutionary pathway of the simian lineage, and observed that the T→C flow was dependent on the local hydrophobic environment of the root AA sequence, while the A→C flow was less dependent on it.

A specific increase of Thr residues in the membrane interior must be presumably connected to some mt functions. Recent studies elucidated that membrane proteins undergo large movements during functions [15], [16]. A trade-off between mobility and stability of proteins may exist [16]. We note that helix–helix interactions play an essential role in the stabilization of membrane proteins [16]–[19] and about half of all helix pairs are formed by classical hydrogen bonds [18], [19]. Interestingly, most of these bonds are found in motifs involving medium-polar residues, such as Thr or Ser. Thr/Ser motifs can also drive the association of model transmembrane helices [28]. Thus, Thr/Ser residues tend to face the membrane interior, the location of which is important for helix–helix interactions, and the methyl (CH_3_) group in Thr is especially suited to the hydrophobic environment of the membrane interior [17]. Consequently, a plausible adaptive explanation for the increase in Thr in the membrane interior is that added Thr reinforced the stability of mt proteins. This Thr increase in adaptive evolution may be an important step toward a solution to the membrane-protein-folding problem [29].

**Figure 6:**
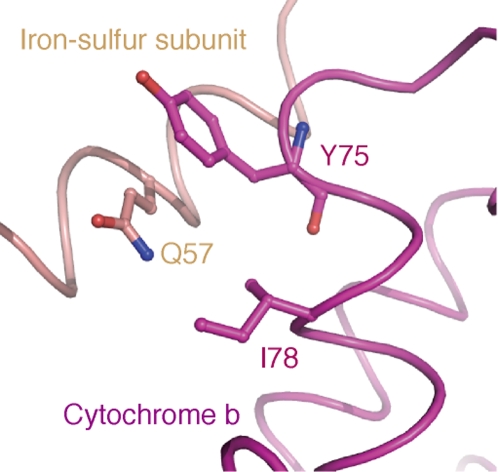
3-dimensional structure around Ile 78 position (helix B) in bovine Cytb. I78, Q57 and Y75 denote three AA positions of Ile 78 (helix B), Gln 57 (helix A) and Try 75 (helix B) in bovine Cytb, respectively.

We searched for AA positions at which putative helix-helix interactions could be formed by the change from Hoa to Thr, on the basis of the bovine crystal structure of respiratory complex III (cytochrome bc1 complex; PDB ID: 1QCR reported by Xia et al. [30]). We first looked at the AA position 78 in Cytb which gives Hoa (such as Ile, Leu, Met, and Val) in most of the 62 taxa of Figure 1. Among the 62 taxa, this position has Thr only in gorilla, baboon, and sperm whale. When Ile78 in the helix B of bovine Cytb is replaced by Thr, the Oγ atom of Thr78 has a possibility of forming a hydrogen bond with the Nε atom of Gln57 in the transmembrane, helix of Rieske iron-sulfur protein (Figure 6). This replacement is of particular interest, because we recently reported that the Ile78Thr replacement in Cytb was more frequently detected in Japanese semi-supercentenarians whose ages were 105 years and over, than in controls [31]. This Ile78Thr replacement in human individuals with extreme longevity may contribute to the stability of the respiratory complex by enhancing the helix-helix interaction within the molecule. The second example is the AA position 180 of Cytb which gives Thr in simians and sperm whale, and Ala in the remaining taxa except for cat with Gly. When Ala180 in helix D of bovine is replaced by Thr, this Thr180 can form a hydrogen bond with the main chain carbonyl group of Ala52 in helix A. The third example is the AA position 302 of Cytb which gives Thr in chimpanzee, orangutan and capuchin of simians, and Hoa in the remaining taxa. When Ala302 in helix F of bovine is replaced by Thr, this Thr302 can form a hydrogen bond with the side chain of Asn114 in helix C. Further detailed molecular simulations and experiments are needed to verify our proposal that the increase of Thr residues in higher primates is associated with an increased stability of mitochondrial proteins but the proposal is supported by our preliminary investigations (Table S2).

To conclude, the possible detection of adaptive evolution in the higher primate lineage gave a hint that the Thr composition in the mt membrane interior is meaningfully correlated with the life span of primates. Future research can establish whether the Hoa→Thr/Ser flow has occurred in a wider range of evolutionary lineages and whether such a flow has a connection to longevity.

## Materials and Methods

### Input data for the present analysis

We obtained complete mt DNA sequences from the NCBI genome database. We used the MLS data from Moosmann and Behl [26], to compare our Thr-MLS correlation model with their Cys-MLS correlation model. We obtained additional MLS data from the Max Planck Institute Longevity Records (http://www.demogr.mpg.de/longevityrecords/) and the AnAge database [34].

### Estimation of ancestral sequences

We used the phylogenetic tree structure (topology) inferred from 62 complete mt AA sequences which was reconstructed via a multidimensional vector space (MVS) method [2]. The AA node sequences were estimated on this tree by applying an extended maximum parsimony method which reduces uncertainty due to convergent evolution [35]. To estimate only the Hoa→Thr flow in Figures 1 and 2, we decomposed the 20 AAs into three groups, Hoa, Thr, and Rma, by disregarding the intra-substitutions within the groups. The node sequences in Figures 3 & 4 and Tables 1 & 2 were estimated by applying the extended maximum parsimony method at the DNA level to the three local lineages of Primates, Cetartiodactyla, and Afrotheria. The resulting DNA sequences were translated into the AA sequences.

### Primary structure analysis and significant test for hydrophobicity dependence of AA and nucleotide flows

The primary structure analysis [21]–[23] for the hydrophobicity dependence of 12 mt proteins involved calculating a moving average, *S*, of the local hydrophobic scores around each AA site by using an orthodox scale model [24]. We estimated the nucleotide flows in two regions (*S*<0.6 and *S*>0.6) of the root sequences, and then determined the significance of the differences between the nucleotide flows in the two regions. In Table 2, we defined n_TC_ and n_CT_ as the numbers of sites of T→C and C→T changes at the second codons in *S*>0.6, respectively, and likewise m_TC_ and m_CT_ as the numbers of sites in *S*<0.6, respectively. We let N and M be the numbers of second codon sites in *S*>0.6 and *S*<0.6, respectively. The significance was calculated by the normalized statistic z = y/V(y)^1/2^, with y = {(n_TC_−n_CT_)/N−(m_TC_−m_CT_)/M}. The variance V(y) of y was calculated as V(y) = V(y_n_)+V(y_m_), with V(y_k_) = V{Q_k,TC_−Q_k,CT_} = {Q_k,TC_(1−Q_k,TC_)+Q_k,CT_(1−Q_k,CT_)+2Q_k,TC_ Q_k,CT_}/K (k and K taking n or m and N or M, respectively). Here, Q_k,L_ = k_L_/K (L takes CT or TC). As a result, the T→C flow in the simian lineage of present interest for adaptive evolution showed a significant hydrophobicity dependence with z = 4.392 and p = 1.123×10^−5^.

### Phylogenetic generalized least-squares

The association between the Thr/Cys compositions and the MLS is partly a result of the shared history that underlies phylogenetic correlations between taxa on the tree structure to be analyzed. This dependence can be estimated by using the phylogenetic generalized least-squares for the regression analysis [27]. We determine constants A_1_, A_2_ and A_3_ by minimizing the function Q = Σ_ij_ (Y_i_−A_1_X_1i_−A_2_X_2i_−A3)W_ij_ (Y_j_−A_1_X_1j_−A_2_X_2j_−A_3_). Here, variables X_1i_ and X_2i_ denote the Thr and Cyr compositions of the *i-th* species, respectively, and Y_i_ denotes the Log {MLS(years)} value of the *i-th* species. The matrix **W** is given by the inverse of **B** whose matrix element B_ij_ is the common ancestral branch length of the *i-th* and *j-th* species. The correlation coefficient R^2^ is estimated by the equation R^2^ = 1−Q/V. Here, V = Σ_ij_ (Y_i_−Y_av_) W_ij_ (Y_j_−Y_av_) with the average value, Y_av_, of Y_i_ (i = 1,…, N).

## Supporting Information

Table S1 Hydrophobicity scores of AAs. Our primary structure analysis used a model of Cowan and Whittaker for hydrophobicity indices of AAs [24]. The * and # symbols denote the hydrophobic AAs (Hoa) defined in this paper and threonine, respectively. Although Trp is hydrophobic, it was not included in Hoa since it did not indicate any flow (Figure 3).
(0.05 MB DOC)

Table S2 The AA sites of mt proteins which changed from Hoa to Thr. This Table lists the AA sites of proteins which changed from the Hoa in the root sequence of primates to the Thr in the chimpanzee or human sequence. These changes took place in the hydrophobic region of S>0.6. The root was defined as the most recent common ancestor of the tree shrew and the primates.
(0.17 MB DOC)

Table S3 The Thr/Cys composition and Log (MLS) values in primates. The second and third columns give the common names of primates and the accession numbers of the DNA sequences in the NCBI database, respectively. The fourth and fifth columns give the Thr and Cys compositions (%) in all AA sites of 12 mt proteins, respectively. The sixth and seventh columns give those in the hydrophobic sites (S>0.6), respectively. The last column gives the Log {MLS (years)} values.
(0.07 MB DOC)
