# Supplementary material for: Adaptive Threonine Increase in Transmembrane Regions of Mitochondrial Proteins in Higher Primates
Source: PLoS One. 2008 Oct 6;3(10):e3343. doi: 10.1371/journal.pone.0003343 (PMC2553178; doi:10.1371/journal.pone.0003343)
Supplement: Table S1 — Hydrophobicity scores of AAs. Our primary structure analysis used a model of Cowan and Whittaker for hydrophobicity indices of AAs [24]. The * and # symbols denote the hydrophobic AAs (Hoa) defined in this paper and threonine, respectively. Although Trp is hydrophobic, it was not included in Hoa since it did not indicate any flow (Figure 3). (0.05 MB DOC) [file pone.0003343.s001.doc]

| **No** | **AAs** | | **hydrophobic score** | |
| --- | --- | --- | --- | --- |
| 1 |  | Ala | 0.35 |  |
| 2 |  | Arg | -1.50 |  |
| 3 |  | Asn | -0.99 |  |
| 4 |  | Asp | -2.15 |  |
| 5 |  | Cys* | 0.76 |  |
| 6 |  | Gln | -0.93 |  |
| 7 |  | Glu | -1.95 |  |
| 8 |  | Gly | 0.00 |  |
| 9 |  | His | -0.65 |  |
| 10 |  | Ile* | 1.83 |  |
| 11 |  | Leu* | 1.80 |  |
| 12 |  | Lys | -1.54 |  |
| 13 |  | Met* | 1.10 |  |
| 14 |  | Phe* | 1.69 |  |
| 15 |  | Pro* | 0.84 |  |
| 16 |  | Ser | -0.63 |  |
| 17 |  | Thr# | -0.27 |  |
| 18 |  | Trp* | 1.35 |  |
| 19 |  | Tyr | 0.39 |  |
| 20 |  | Val* | 1.32 |  |
