# Supplementary material for: Adaptive Threonine Increase in Transmembrane Regions of Mitochondrial Proteins in Higher Primates
Source: PLoS One. 2008 Oct 6;3(10):e3343. doi: 10.1371/journal.pone.0003343 (PMC2553178; doi:10.1371/journal.pone.0003343)
Supplement: Table S2 — The AA sites of mt proteins which changed from Hoa to Thr. This Table lists the AA sites of proteins which changed from the Hoa in the root sequence of primates to the Thr in the chimpanzee or human sequence. These changes took place in the hydrophobic region of S>0.6. The root was defined as the most recent common ancestor of the tree shrew and the primates. (0.17 MB DOC) [file pone.0003343.s002.doc]

| **Proteins** |  | **Site No** |  | **Root** | **Chimpanzee** | **Human** |
| --- | --- | --- | --- | --- | --- | --- |
| ND1 | 1 | 69 |  | L | T | T |
|  | 2 | 73 |  | I | T | T |
|  | 3 | 87 |  | I | T | A |
|  | 4 | 108 |  | I | T | T |
|  | 5 | 153 |  | I | T | T |
|  | 6 | 236 |  | A | T | T |
|  | 7 | 261 |  | I | T | T |
|  | 8 | 309 |  | I | T | I |
|  | 9 | 310 |  | L | T | T |
| ND2 | 1 | 15 |  | L | T | A |
|  | 2 | 29 |  | I | T | T |
|  | 3 | 156 |  | M | T | T |
|  | 4 | 206 |  | M | T | T |
|  | 5 | 213 |  | M | T | T |
|  | 6 | 214 |  | A | T | T |
|  | 7 | 247 |  | M | T | T |
|  | 8 | 286 |  | A | T | T |
| Cox2 | 1 | 45 |  | M | T | T |
| ATP8 | 1 | 11 |  | I | T | T |
|  | 2 | 14 |  | V | T | T |
|  | 3 | 24 |  | L | T | T |
| ATP6 | 1 | 53 |  | I | T | T |
|  | 2 | 135 |  | I | T | T |
|  | 3 | 189 |  | A | A | T |
|  | 4 | 194 |  | I | T | T |
| Cox3 | 1 | 40 |  | I | T | M |
|  | 2 | 44 |  | A | T | M |
|  | 3 | 88 |  | I | T | T |
| ND3 | 1 | 9 |  | I | T | I |
|  | 2 | 21 |  | A | T | T |
| ND4L | 1 | 13 |  | I | T | T |
|  | 2 | 48 |  | A | T | T |
|  | 3 | 51 |  | I | T | T |
|  | 4 | 62 |  | I | T | A |
| ND4 | 1 | 36 |  | L | T |  |
|  | 2 | 75 |  | M | T | T |
|  | 3 | 123 |  | A | T | T |
|  | 4 | 166 |  | M | T | T |
|  | 5 | 199 |  | M | T | T |
|  | 6 | 298 |  | V | T | T |
|  | 7 | 390 |  | L | T | I |
|  | 8 | 395 |  | M | T | T |
|  | 9 | 454 |  | L | T | T |
| ND5 | 1 | 11 |  | I | T | T |
|  | 2 | 40 |  | A | T | T |
|  | 3 | 49 |  | M | T | T |
|  | 4 | 85 |  | M | T | M |
|  | 5 | 207 |  | F | T | P |
|  | 6 | 273 |  | I | T | T |
|  | 7 | 370 |  | M | T | T |
|  | 8 | 426 |  | A | T | T |
|  | 9 | 428 |  | M | T | T |
|  | 10 | 496 |  | I | T | T |
|  | 11 | 594 |  | V | T | T |
| ND6 | 1 | 150 |  | A | T | T |
| Cytb | 1 | 46 |  | V | T | T |
|  | 2 | 122 |  | A | T | A |
|  | 3 | 123 |  | A | T | T |
|  | 4 | 180 |  | A | T | T |
|  | 5 | 190 |  | A | T | A |
|  | 6 | 191 |  | A | A | T |
|  | 7 | 193 |  | V | T | A |
|  | 8 | 194 |  | F | T | A |
|  | 9 | 241 |  | I | T | T |
|  | 10 | 243 |  | V | T | T |
|  | 11 | 279 |  | A | T | T |
|  | 12 | 302 |  | A | T | A |
|  | 13 | 330 |  | A | T | A |
|  | 14 | 348 |  | I | I | T |
|  | 15 | 349 |  | I | T | I |
|  | 16 | 360 |  | I | T | T |
|  | 17 | 367 |  | L | I | T |
