# Supplementary material for: Adaptive Threonine Increase in Transmembrane Regions of Mitochondrial Proteins in Higher Primates
Source: PLoS One. 2008 Oct 6;3(10):e3343. doi: 10.1371/journal.pone.0003343 (PMC2553178; doi:10.1371/journal.pone.0003343)
Supplement: Table S3 — The Thr/Cys composition and Log (MLS) values in primates. The second and third columns give the common names of primates and the accession numbers of the DNA sequences in the NCBI database, respectively. The fourth and fifth columns give the Thr and Cys compositions (%) in all AA sites of 12 mt proteins, respectively. The sixth and seventh columns give those in the hydrophobic sites (S>0.6), respectively. The last column gives the Log {MLS (years)} values. (0.07 MB DOC) [file pone.0003343.s003.doc]

| ID No. |  | accession No. | Cys (%) | Thr (%) | Cys in S>0.6 (%) | Thr in S>0.6 (%) | Log {MLS(years)} |
| --- | --- | --- | --- | --- | --- | --- | --- |
| 1 |  | NC_002763 | 0.581 | 9.715 | 0.695 | 7.650 | 3.784 |
| 2 |  | NC_007009 | 0.582 | 10.735 | 0.649 | 9.805 | 3.434 |
| 3 |  | NC_006901 | 0.660 | 9.398 | 0.816 | 7.682 | 3.178 |
| 4 |  | NC_010299 | 0.687 | 9.823 | 0.786 | 8.357 | 3.190 |
| 5 |  | NC_010300 | 0.766 | 8.395 | 0.672 | 7.666 | 3.401 |
| 6 |  | NC_001645 | 0.554 | 9.158 | 0.600 | 8.467 | 3.989 |
| 7 |  | NC_002082 | 0.528 | 9.422 | 0.551 | 7.581 | 3.689 |
| 8 |  | NC_004025 | 0.713 | 8.137 | 0.669 | 7.358 | 3.296 |
| 9 |  | NC_005943 | 0.712 | 10.343 | 0.704 | 8.099 | 3.555 |
| 10 |  | NC_002764 | 0.660 | 10.135 | 0.645 | 8.309 | 3.091 |
| 11 |  | NC_008216 | 0.634 | 9.081 | 0.667 | 7.205 | 3.045 |
| 12 |  | NC_002765 | 0.712 | 8.654 | 0.557 | 7.247 | 3.296 |
| 13 |  | NC_001644 | 0.581 | 9.580 | 0.612 | 9.388 | 3.912 |
| 14 |  | NC_001643 | 0.528 | 9.554 | 0.614 | 9.004 | 4.094 |
| 15 |  | NC_001992 | 0.607 | 10.451 | 0.632 | 9.059 | 3.611 |
| 16 |  | NC_001646 | 0.633 | 9.739 | 0.752 | 8.681 | 4.078 |
| 17 |  | NC_008217 | 0.581 | 8.815 | 0.805 | 7.651 | 2.773 |
| 18 |  | NC_008220 | 0.607 | 9.213 | 0.754 | 7.682 | 3.219 |
| 19 |  | NC_008218 | 0.608 | 8.721 | 0.720 | 7.204 | 3.384 |
| 20 |  | NC_006900 | 0.581 | 9.240 | 0.746 | 7.593 | 3.219 |
| 21 |  | NC_002521 | 0.766 | 8.056 | 0.893 | 6.799 | 2.303 |
| 22 |  | NC_001807 | 0.581 | 9.139 | 0.616 | 8.140 | 4.808 |
